# Bronchial Artery Embolization in Life-Threatening Massive Hemoptysis

**DOI:** 10.5812/ircmj.16618

**Published:** 2013-12-05

**Authors:** Hossein Ghanaati, Ali Shakouri Rad, Kavous Firouznia, Amir Hossein Jalali

**Affiliations:** 1 Department of Radiology, Advanced Diagnostic and Interventional Radiology Research Center (ADIR), Tehran University of Medical Sciences, Tehran, IR Iran; 2 Department of Radiology, Sina Hospital, Tehran University of Medical Sciences, Tehran, IR Iran

**Keywords:** Hemoptysis, Computed Tomography, Angiography, Bronchial Artery Embolization

## Abstract

**Background:**

Massive hemoptysis is a potentially life threatening respiratory emergency and mandates immediate investigation and intervention. There is no universal consensus regarding the optimal management of these patients, and there are no large series of patients studied.

**Objectives:**

Here we reported thirty Iranian patients who were managed with bronchial artery embolization.

**Patients and Methods:**

All the patients had already been assessed by computerized tomography (CT) to localize and delineate the underlying etiology except 2 patients who had not undergone CT scan.

**Results:**

Tuberculosis, bronchiectasis, and lung cancer/metastasis were the most common causes, detected in 14(47%), 5(17%) and 4(13%) patients respectively. Other causes of hemoptysis including chronic bronchitis, interlobar artery aneurysm, hydatid cyst, arteriovenous fistula, pulmonary embolism, and exposure to chemical weapons each detected in one patient separately. All of them had abnormal chest CT scans, except for 2 patients who had not undergone CT scan (one with hydatid cyst and another with bronchial tumor diagnosed with bronchoscopy). Bleeding location which has been confirmed with angiography could be predicted with CT scan among 7 of 14 patients with TB, (sensitivity=50%). While this rate was 100% among all other patients with other diagnosis who had undergone CT scan.

**Conclusions:**

In conclusion complementary to the previous studies our results have demonstrated that bronchial artery embolization remains as one of the most efficient procedures in managing massive hemoptysis, with minimal rate of complications.

## 1. Background

Massive hemoptysis is a potentially life-threatening respiratory emergency mandating immediate survey and management. Comparatively, it is almost a common presentation in practice (1). It is defined as expectoration of 300 to 600 mL of blood in a period of 24 hours (2). The most common etiologies include bronchiectasis, cystic fibrosis, neoplasm, sarcoidosis, tuberculosis, and other infections (3, 4). While only five percent of hemoptysis is massive, some studies report a mortality rate of up to 80 percent in this subgroup, mainly due to asphyxiation (5).

There is no global consensus regarding the optimal management of these patients, and there are no large series of patients studied (5). Medical or surgical management for massive hemoptysis mainly are ineffectual, with a mortality rate of 35%-100%. Embolization has an initial success rate of 95 percent, with less morbidity and mortality than surgical resection (3). As a result, transcatheter bronchial artery embolization (BAE) has become treatment of choice for massive hemoptysis, while surgical approach remains a complementary management for embolization failure or recurrent massive hemoptysis after multiple embolizations (5).

## 2. Objectives

Embolization as a treatment for hemoptysis is recently developed in Iran. Data on its feasibility and effectiveness are scarce from our region. Hereby, we decided to report our preliminary experience with a series of 30 hempotytic Iranian patients who underwent transcatheter embolization.

## 3. Patients and Methods

From 2001 may to 2003 march, 30 patients with moderate or severe hemoptysis who had been referred for transcatheter embolization to Medical Imaging Center of Imam Khomeini hospital in Tehran, Iran were included. All the patients had already been assessed by computerized tomography (CT) to localize and delineate the underlying etiology except 2 patients who had not undergone CT scan.

At first, in angiography unit, non-selective aortograms followed by selective bronchial arteriograms were obtained using 4F Cobra catheters. If the results of the previous evaluations were not diagnostic, non-bronchial systemic arteries including subclavian and intercostal arteries were studied using selective 4F glide catheterization. Spinal arteries were identified during angiography in all patients, since these arteries may have common origins with intercostal branches. The identical catheters which were applied to study bronchial angiography were utilised in bronchial artery embolization. Polyvinyl alcohol particles (PVA) >250 microns were used in all the cases for bronchial artery embolization. The mean post-interaction hospitalization was 2 days (ranging from 12 hours to 8 days) for observing and supportive managing of patients until being clinically stable. The angiographic arrangement (GE DEX DSA, GE Healthcare) was set to 1200 mA and 140 kVp.

## 4. Results

The Demographics revealed twenty five patients (83%) were male. The mean age of cases was 55, ranging from 42 to 83. Tuberculosis (TB), bronchiectasis, and lung cancer/metastasis were the most common causes of hemoptysis detected in 14 (47%), 5 (17%) and 4 (13%) patients respectively. Other causes of hemoptysis including chronic bronchitis, interlobar artery aneurysm, hydatid cyst, arteriovenous fistula, pulmonary embolism, and exposure to chemical weapons each detected in one patient separately. One patient had no possible diagnosis. All of the patients had abnormal chest CT scans, except for 2 patients who had not undergone CT scan (one with hydatid cyst and another with bronchial tumor diagnosed with bronchoscopy). Location of the lesion/hemorrhage which has been confirmed with angiography could be predicted with CT scan among 7 of 14 patients with TB, (sensitivity = 50%). CT scan was diagnostic for the location of hemorrhage among all other patients with other diagnosis who had undergone CT scan. Locations of hemorrhage according to the angiographic findings are summarized in Table 1. 

**Table 1.** Location of Hemorrhage Among the Patients with Hemoptysis According to Angiography

	Right		Left		Both Side		No, Blush
	Apical	Others	Apical	Others	Apical	Others	
**Tuberculosis**	5	1 (posterior segment of upper lobe)	4		2		2
**Bronchectasis**	3	1 (area of bronchial artery)				1 (bronchial arteries)	
**Lung cancer**	1	2(area of bronchial artery)		1 (area of bronchial artery			

The location of hemorrhage in patients with chronic bronchitis, inter-lobar artery aneurysm, hydatid cyst, arteriovenous fistula, pulmonary embolism, and exposure to chemical weapons were left apical segment for the first one and area of right bronchial artery for the others.

Acute episodes of hemoptysis were controlled by BAE in all the patients. None of the patients had any serious complication including spinal cord ischemia, pulmonary infarction, bronchial wall necrosis , referral pain to the ipsilateral orbital and frontal area, bronchoesophageal fistula, ischemic colitis, ischemic myelopathy and transient cortical blindness. Although 25 of patients (83%) complained from a mild to moderate chest pain.

## 5. Discussion

Since 1973 BAE has been considered as an effective approach in managing massive hemoptysis (6-8). Due to the presence of severe bilateral pulmonary disease and other medical comorbidities, most of these patients are not a good candidate for surgical intervention (9). Since the considerable mortality rate of conservative and the emergent surgical approach in massive hemoptysis which are 40% (10) 10 and 50% to 100% respectively (11), BAE remains the most effective intervention in managing massive and recurrent hemoptysis (12).

As it was discussed above, Polyvinyl alcohol particles (PVA) >250 microns were applied in all the cases for bronchial artery embolization. Usage of coils in BAE is not proper because of the proximal occlusion and limitation in repeat embolization (13, 14). Large embolization is not preferred in BAE either, due to predisposing rapid distal collateral formation and limiting second intervention through blocking the access to embolization area (15). In contrast the passage of fine particles through bronchopulmonary agents results in ischemia in pulmonary tissue; Hence the prohibition of the usage of Particles <250 microns (15). In contrast to gelatin sponge particles, Polyvinyl chloride leads to permanent occlusion (13). However the disadvantages of PVA include catheter occlusion and being radiolucent (16).

Massive hemoptysis has numerous etiologies, of which the frequency differs between the Western and non-Western societies (17). In non-Western world like Iran, pulmonary tuberculosis remains the most common etiology for massive hemoptysis (18). While, bronchogenic carcinoma and chronic inflammatory lung diseases constitute the most common cause in Western countries (17). Other causes include Lung abscess, coagulopathy, disseminated intravascular coagulation, pulmonary embolism, bullous emphysema, pulmonary hypertension, goodpasture's syndrome, wegener's granulomatosis, mitral stenosis, tricuspid endocarditis, congenital heart disease, pulmonary artery aneurysm (Rasmussen aneurysm), hydatid cyst, arteriovenous fistula (19), mediastinal teratomas (20), Primary mediastinal haemangiopericytoma (21), transthoracic fine needle aspiration (TFNA) biopsy, (22) and exposure to chemical weapons (23).

The role of bronchoscopy and CT scan have been evaluated and compared in detecting the location of bleeding and the cause of bleeding (24). According to Revel et al. (24) CT scan is more efficient in identifying the underlying cause of massive hemoptysis. Multidetector row computed tomography (MDCT) is a useful tool in identifying the anatomical feature of bronchial artery and evaluating the need of BAE in hemoptysis (25). MDCT also can demonstrate bronchial and non-bronchial artery in hemoptysis (26). In contrast to conventional chest radiography which may show a normal lung in cases of malignancy (27), CT scan has a proven diagnostic value in determining the cause and location of bleeding (28, 29).

Among those with a normal chest radiograph, HRCT is diagnostic in 30% of patients while fiber-optic bronchoscopy (FOB) demonstrates the underlying cause in 10% of patients (30). In addition, CT reveals the cause of hemoptysis in 50% of patients in whom FOB findings are normal (31). So a CT scan is essential in all patients with hemoptysis before taking FOB even if the chest X-ray is normal (30), also CT and FOB are complementary to each other and not competitive (29). CT can also detect the site of bleeding in 63%–100% of patients with hemoptysis, which has a greater rate compared to FOB (28, 29, 32). The modern CT scan shortens the time of scanning and makes the whole process of scanning in critically ill patients feasible. Contrast-enhanced CT helps us with identifying the involved vessel (32). The relatively lower sensitivity of CT in localization of hemorrhage foci in TB might be due to diffused nature of lung involvement in this disease.

In the case of malignancy or conditions such as sarcoidosis, PET scan is a useful non-invasive diagnostic imaging modality (4, 33-36).

Bronchial arteriography and embolization were well-tolerated by our patients. An immediate control of bleeding was achieved with embolization in all patients. Our results are similar to those of a study by Ustunsoz et al. (37) who reported that among 10 patients undergoing attempted embolization procedures for hemoptysis, the immediate control of bleeding was achieved in all cases. Cremaschi et al. (38) evaluated 209 patients who had been embolized for hemoptysis and noted that immediate control was achieved after BAE in 205 (98%). Rabkin et al. (39) evaluated 306 patients and found that BAE controlled acute bleeding in 278 (91%).

Several complications are associated with BAE. The most common complication of BAE is chest pain, reported in 24%-91% (12). One of the catastrophes in BAE is spinal cord ischemia caused by spinal artery occlusion occurring in 1.4%-6.5% of cases (12). Spinal and bronchial arteries were not originating from the same truncus in any of the angiograms of our patients. However, as Poyanlı et al. previously reported, origination of the spinal and bronchial arteries from the same truncus is not a contraindication for BAE (2) because particles >250 microns are large enough to not occlude spinal arteries distally (40). None of the patients in the present study had any of the complications reported in the literature and mentioned above. This might be due to the limited number of patients in the study. Several rarely occurring complication have been previously reported, including pulmonary infarction, bronchial wall necrosis , referral pain to the ipsilateral orbital and frontal area, bronchoesophageal fistula, ischemic colitis, ischemic myelopathy and transient cortical blindness (32), meanwhile these complication are rare if the arteriographers are experienced (41, 42). With the increase in BAE experience, the complication rate has declined during the recent years (14).

In conclusion complementary to the previous studies our results have demonstrated that BAE remains as one of the most efficient procedure to stabilize massive hemoptysis and definitely treating some of them without any severe complications, especially in the hands of an expert and skillful agent.
